# Exploration of country-specific barriers and facilitators for the implementation of physical activity according to the EULAR physical activity recommendations for people with rheumatic musculoskeletal diseases in four different European countries: the COPA project

**DOI:** 10.1007/s10067-026-07984-5

**Published:** 2026-02-16

**Authors:** Özgül Öztürk, David Ueckert, Leti van Bodegom-Vos, Salima van Weely, Özlem Feyzioğlu, Karin Niedermann, Anne-Kathrin Rausch Osthoff, Thomas Davergne

**Affiliations:** 1 Department of Physiotherapy and Rehabilitation, Acıbadem Mehmet Ali Aydınlar University, Kayışdağı St, No:32, 34752 Ataşehir, Istanbul Turkey; 2 Department of Physiotherapy, Leiden University Medical Center, Leiden, Netherlands; 3 Department of Biomedical Data Sciences, Leiden University Medical Center, Leiden, Netherlands; 4 Institute of Allied Health Professions, HU University of Applied Sciences, Utrecht, Netherlands; 5 Department of Orthopedics, Rehabilitation and Physical Therapy, Leiden University Medical Center, Leiden, Netherlands; 6 School of Health Sciences, Institute of Physiotherapy, Zurich University of Applied Sciences, Zurich, Switzerland; 7 Inserm, INRAE, Center for Research in Epidemiology and Statistics (CRESS), Université Paris Cité and Université Sorbonne Paris Nord, 75004 Paris, France

**Keywords:** Barrier, Facilitator, Implementation, Inflammatory arthritis, Osteoarthritis, Physical activity

## Abstract

**Objectives:**

Promotion of physical activity (PA) in individuals with rheumatic and musculoskeletal diseases (RMDs) is essential for disease management, yet evidence on social, environmental, and system-level determinants remains limited. This study aimed to quantify the prevalence of these determinants and compare them across four European nations.

**Method:**

A cross-country survey was developed based on a scoping review and semi-structured stakeholder interviews. The survey comprised 27 items across social, environmental, and system domains. Participants rated each item as a facilitator, barrier, or neutral, using a scale from − 10 (barrier) to + 10 (facilitator). Responses were analyzed to assess cross-country differences in demographic characteristics, PA behavior, and determinant ratings.

**Results:**

A total of 734 individuals with RMDs participated (41.1% RA, 40.7% axSpA, and 18.1% OA) from France (30.5%), Switzerland (34.4%), the Netherlands (17.3%), and Turkey (17.7%). Significant between-country differences were identified in PA behaviors and demographics (*p* < 0.05). Overall determinant scores did not differ significantly (*p* = 0.101). Key facilitators varied across countries: “knowledge and fitness to perform exercises” was prominent in Switzerland; “scheduled exercises” in the Netherlands and France; and “health professionals” in France and Turkey. Common barriers included “weather conditions”—particularly in Turkey and the Netherlands—“costs of memberships or sport facilities,” especially in France, and work-related duties in Turkey and the Netherlands.

**Conclusions:**

Despite comparable overall scores, the relevance of social, environmental, and system-level determinants of PA varied across countries These findings highlight the importance of country-specific contextual factors for understanding PA participation and for designing tailored, effective PA promotion strategies in people with RMDs.

**Table:** 

**Key Points**
• *This study provides novel cross-country insights by comparing contextual determinants across four European countries on how cultural norms and environmental infrastructures shape PA behavior in people with RMDs*. • *Context tailored promotion strategies may be crucial to increase adherence to PA recommendations and to make the implementation of policies more effective*.

**Supplementary Information:**

The online version contains supplementary material available at 10.1007/s10067-026-07984-5.

## Introduction

Rheumatic and musculoskeletal diseases (RMDs) are a leading cause of disability worldwide, with rheumatoid arthritis (RA), axial spondyloartritis (axSpA), and osteoarthritis (OA) among the most common and increasingly prevalent forms [1–3]. Physical activity (PA) is recognized as a key strategy for improving symptoms and health outcomes in individuals with RMDs, including the management of comorbidities [4]. PA offer multiple benefits, in terms of function, pain, psychological well-being, and disease-related outcomes in individuals with RMDs [5–7]. Current evidence supports general PA recommendations as both effective and safe, being an essential component of disease management for people living with RA, axSpA, and OA [6, 8].

Despite the well-established benefits of PA, individuals with RMDs continue to show insufficient PA adherence to PA recommendations, as evidenced by both objective and subjective assessments [9–11]. The European Alliance of Associations for Rheumatology (EULAR) recommendations highlight that in order to effectively promote PA in RMDs, both general and disease-specific barriers and facilitators need to be systematically identified and addressed [6]. Research has shown that disease-specific or personal barriers—such as pain, fatigue, reduced physical function, psychological distress, or fear of exacerbating symptoms—are particularly influential in limiting PA participation [12–14] and several questionnaires have been developed to systematically assess these factors [15, 16]. However, the systematic evaluation of extra-personal factors, such as environmental, social, organizational, and systemic barriers and facilitators to PA in RMDs underexplored.

The EULAR-IMPACT consortium underlined that effective implementation requires addressing determinants at both the organizational and healthcare system levels [17]. Although a general trend shows insufficient PA among individuals with RMDs, disparities seem to exist between countries [18]. For example, access to health professionals, educational status of individuals, and transport infrastructure of countries may vary across countries [19]. These differences point out the need to systematically assess and identify facilitators and barriers on the social-environmental level and the local-system level. Although frameworks such as the Theoretical Domains Framework (TDF) [20] exist to guide such assessments, no questionnaires for RMD populations or systematic cross-county evaluations have yet been conducted specifically for extra-personal determinants (also referred to as extra-personal in this study). Understanding these factors is essential to induce sufficient PA engagement ensuring the effective implementation of PA recommendations [6].

Thus, the primary objective of this study is to quantify the prevalence of extra-personal barriers and facilitators to PA and to compare the data across four European countries. We hypothesized that the survey would identify distinct country-specific determinants that influence adherence to PA behavior within these contextual domains.

## Materials and methods

### Design

A cross-sectional observational study was performed in four European countries (France, Switzerland, the Netherlands, and Turkey). Ethical approval was obtained from each of the contributing country (France URPS2024-A01933-44, Switzerland BASEC 2024–01274, the Netherlands W24.003, and Turkey ATADEK 2024–12/527. The present study was conducted in accordance with the principles of the Declaration of Helsinki. In addition, the study followed the Checklist for Reporting Results of Internet E-Surveys (CHERRIES) guidelines [21].

### Questionnaire development

Since no validated questionnaire addressing this topic was available, a new instrument was developed named as COPA-BFQ (Country-specific Physical Activity Barriers and Facilitators Questionnaire). A preliminary list of potential social, environmental, and system-level determinants barriers and facilitators to PA was generated from a previous scoping review [22]. This list was then refined through semi-structured interviews conducted in each participating country with an equal number of patients (*n* = 4), physical therapists (*n* = 2), and one rheumatic diseases expert. All 28 interviewees were asked to evaluate whether each item was relevant, clearly formulated, and comprehensive, and were encouraged to suggest additional items if they perceived any missing points. Items that were considered relevant by at least half of the stakeholders were retained. Based on these items, the project team prepared the initial English version of the survey, which was reviewed and approved for translation into four languages. To ensure linguistic clarity and accuracy, the English version of the questionnaire was further reviewed by a native English speaker.

The domains of the questionnaire were inspired by the TDF [20] and comprised three domains: social (7 items), environmental (13 items), and system (7 items). It included 27 items, each rated as a facilitator, barrier, or neutral for participation in PA. If rated as a facilitator or barrier, the strength of the item’s impact could be scored from 0 to 10, as proposed in previous questionnaire [15]. A higher score indicated that the item was considered more of a facilitator, and vice versa for barriers. Adaptive questioning was used so that the scoring section for each item was displayed only if the participant first rated that item as a facilitator or a barrier.

The questionnaire is available in the Supplementary File 1. The TRAPD [23] approach was used to translate the COPA into the four languages used for national surveys. Items were translated from English by a both expert in the field and a professional agency in each country. Following the TRAD approach, no backward translation was performed. Discrepancies between versions were resolved collaboratively to ensure linguistic validity and consistency. The project team also ensured that terms and definitions were appropriately translated and understandable. Preliminary testing was conducted with 8 patients (2 per country), who assessed item fluency.

Additional to barriers and facilitators, participants were asked how often they perform physical activity or strength training per week. Further, demographics and years since diagnosis were recorded.

### Data collection

The survey was administered in France, Switzerland, the Netherlands, Turkey, and between August 2024 and April 2025. The questionnaire was filled digitally via survey systems; however, initial contact was made on the internet and face-to-face. Each country aimed to recruit at least 100 patients. The target of recruiting approximately 100 participants per country was determined a priori based on pragmatic and methodological considerations related to the exploratory aims of this study. As the primary objective was to pilot-test the newly developed COPA questionnaire and to assess feasibility, item distributions, and preliminary domain and total scores across countries, a formal sample size calculation was not performed. Sample sizes of this magnitude are commonly used in exploratory and pretest questionnaire studies to obtain stable descriptive estimates and to identify items with limited variability or potential measurement issues. Participation was voluntary, and consent could be withdrawn at any time by exiting the survey. Patients were recruited from rheumatology departments, ongoing studies, social media, and national patient associations. Participants were included if they were above 18 years of age, had a self-confirmed diagnosis of RA, axSpA or OA, and ability to read and write in the language of the participating country. No incentives of any kind—monetary, material, or non-monetary—were offered to participants for completing the survey. The number of pages for the questionnaire differed for each country. The respondents could not review their answers before submitting.

### Statistical analysis

Data were exported from the national secure survey platforms into Excel (Microsoft Corp., Redmond, WA) and were analyzed using SPSS 21.0 (IBM Corporation, Armonk, New York). Only fully completed questionnaires were included in the analysis; partially completed or early terminated responses were excluded. No personal data were collected or stored. Entries were hand-checked to ensure data integrity, rather than using automated duplicate-detection procedures. Frequencies and number of patients were reported for categorical variables and means with standard deviations or medians with interquartile ranges were reported for continuous variables. A global score ranging from −10 (strong barrier) to 10 (strong facilitator) was calculated, with 0 indicating neutrality for each item. An overall score is calculated by adding up all the items, ranging from − 270 to + 270. Additionally, subdomain scores were computed for each domain (social, environmental, and system). The Shapiro–Wilk test, Q-Q plots, and histograms were used to check the normality of the data. Cross-country comparisons were performed with one-way ANOVA or Kruskal–Wallis tests based on the distribution of data, followed by appropriate post hoc analyses (Dunn-Bonferroni or Tamhane). Chi-square or Fisher’s exact test was used for categorical comparison. Figures were produced using Python (version 3.13.7; Python Software Foundation). Data processing and statistical calculations were performed in pandas and NumPy, while visualizations were generated using matplotlib.

## Results

A total of 734 individuals with RMDs completed the questionnaire. Data were missing for age (*n* = 47) and gender (*n* = 39) due to an omission in an earlier version of the evaluation form, which was corrected after detection. The remaining 687 participants had a mean age of 57.0 years (standard deviation (SD) = 12.8), and the majority was female (*n* = 578, 83.2%).

Table 1 summarizes the demographic data and data related to PA behavior. The distributions of age, gender, type of diagnosis, time since diagnosis, and the number of days spent in PA and strength training were examined. All parameters differed significantly between countries (*p* < 0.001), with the least strength training in Turkey, and more strength training days in Switzerland than France (*p* = 0.001). Days spent for strength training did not differ between the Netherlands, and France, and Switzerland (*p* > 0.05). Additionally, pairwise comparisons revealed significant differences in PA days across countries (*p* < 0.05). Participants in France were significantly older than those in Switzerland, the Netherlands, and Turkey (*p* ≤ 0.03). Participants in Turkey had a more recent diagnosis compared with participants in France and Switzerland (*p* ≤ 0.001). In additional, participants in France were more recently diagnosed than those in Switzerland and the Netherlands (*p* < 0.05).

**Table 1:** Comparison of variables among people with RMDs across countries

Variables	All patients (*n* = 734)	France (*n* = 224)	Netherlands (*n* = 127)	Switzerland (*n* = 253)	Turkey (*n* = 130)	*p* value
Age, mean (SD)	57.03 (12.84)	60.71 (12.05)	56.11 (11.71)	56.17 (13.69)	52.95 (11.56)	** < 0.001**†
Gender, female, n (%)	578 (83.2)	206 (92.0)	114 (89.8)	158 (73.8)	100 (76.9)	** < 0.001**
Years since diagnosis, mean (SD)	10 (4–20)	15 (6–26)	8 (3–17)	10 (4–24)	7 (3–12)	** < 0.001††**
Physical activity days per week, median (IQR)	3 (2–5)	3 (1–4)	3 (2–5)	4 (3–6)	2 (0–3)	** < 0.001** ††**†**
Strength training days per week, median (IQR)	1 (0–2)	1 (0–2)	1 (0–2)	1 (0–3)	0 (0–0)	** < 0.001††††**
*Diagnosis, n (%)*						** < 0.001**
Osteoarthritis	133 (18.1)	39 (17.4)	24 (18.0)	29 (11.5)	41 (31.5)	
Rheumatoid arthritis	302 (41.1)	147 (65.6)	62 (46.6)	40 (15.8)	53 (40.8)	
Axial spondyloarthritis	299 (40.7)	38 (17.0)	41 (30.8)	184 (72.7)	36 (27.7)	
COPA-BFQ total score, mean (SD)	40.04 (45.14)	41.06 (49.30)	37.48 (44.30)	44.22 (35.93)	32.61 (53.28)	0.101
Social, mean (SD)	16.08 (13.60)	18.35 (13.99)	15.88 (13.42)	14.72 (11.73)	15.00 (15.93)	0.022*
Environmental, mean (SD)	21.55 (27.05)	20.91 (30.51)	22.29 (25.22)	25.54 (21.83)	14.15 (30.12)	0.001**
System, median (IQR)	0 (− 4–10)	0 (− 5–10)	0 (− 8–7)	5 (0–10.5)	0 (− 7.25–10)	0.006***

Bold values indicate significancy. *SD* standard deviation, *IQR* inter-quartile range. Post hoc analysis was performed by using Dunn-Bonferroni or Tamhane tests

†Significant difference between Switzerland, the Netherlands, Turkey, and France

††Significant difference between Switzerland, France, and Turkey, and also between Netherlands, Switzerland, and France

†††Pairwise comparisons demonstrated significant differences between each country

^††^^**††**^Pairwise comparisons demonstrated significant differences between each country except for the Netherlands, and France and Switzerland

*Signfificant difference between France and Switzerland

**Significant difference between Switzerland and Turkey

***Significant difference between Switzerland and Netherlands

**Fig. 1:**
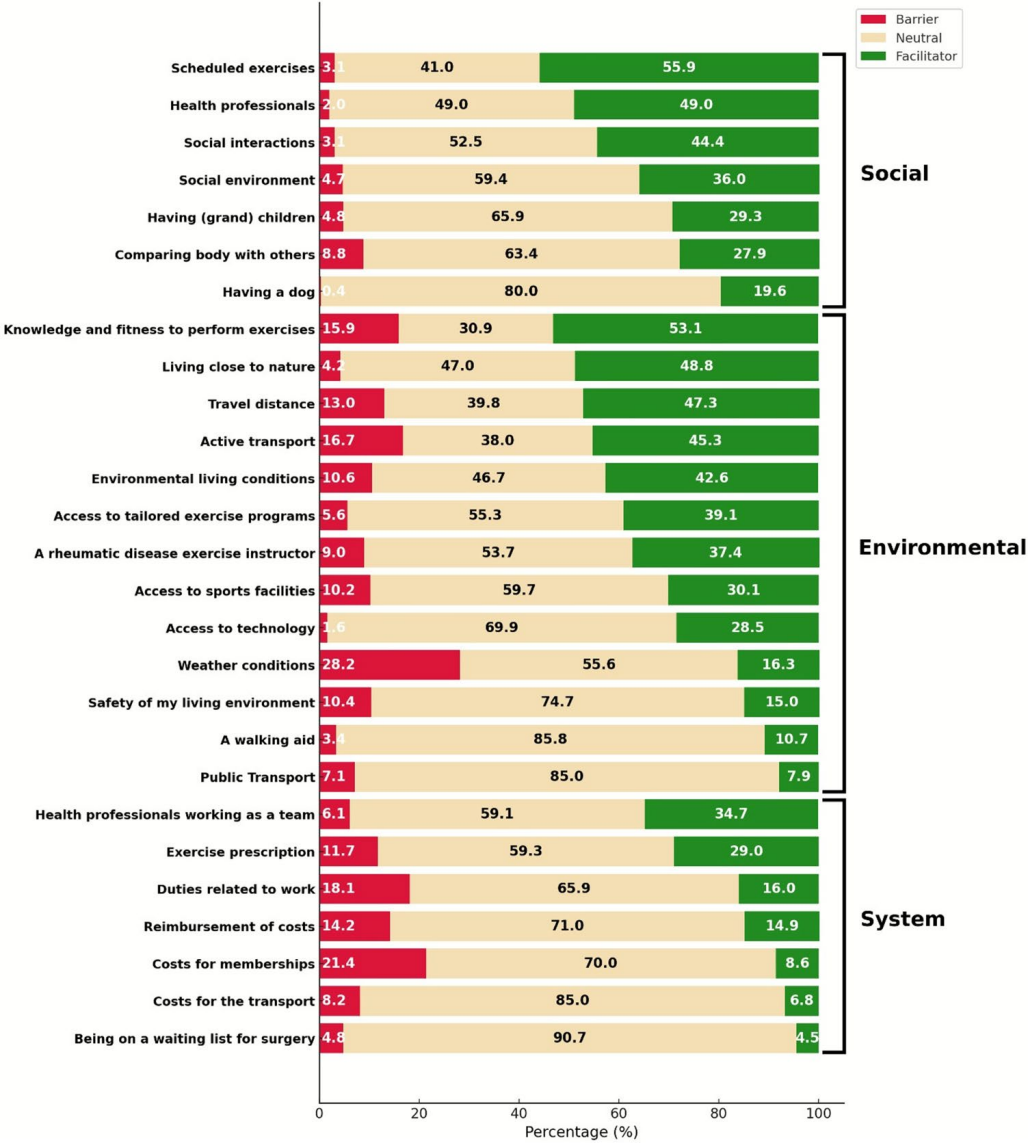
Percentage distribution of perceived barriers, neutral factors, and facilitators influencing physical activity across the social, environmental, and system domains of the COPA-BFQ

Figure 1 presents 27 items with related proportion of participants rating them as barrier, neutral, or facilitator, organized in three sub-categories. Among social items, “scheduled exercises” were most frequently reported as a facilitator. Within environmental items, “knowledge and fitness to perform exercises” was a key facilitator, whereas “weather conditions” emerged as a major barrier. For system-related items, “health professionals working as a team” was the most common facilitator, while “costs for membership to sport facilities or equipment” and “work-related duties” were predominantly considered barriers.

The overall questionnaire score did not significantly differ between countries (*p* = 0.101) (Table 1). However, sub-category analyses revealed higher social scores in France compared to Switzerland (*p* = 0.015), higher environmental scores in Switzerland compared to Turkey (*p* = 0.001), and higher system-related scores in Switzerland compared to the Netherlands (*p* = 0.007).

**Fig. 2:**
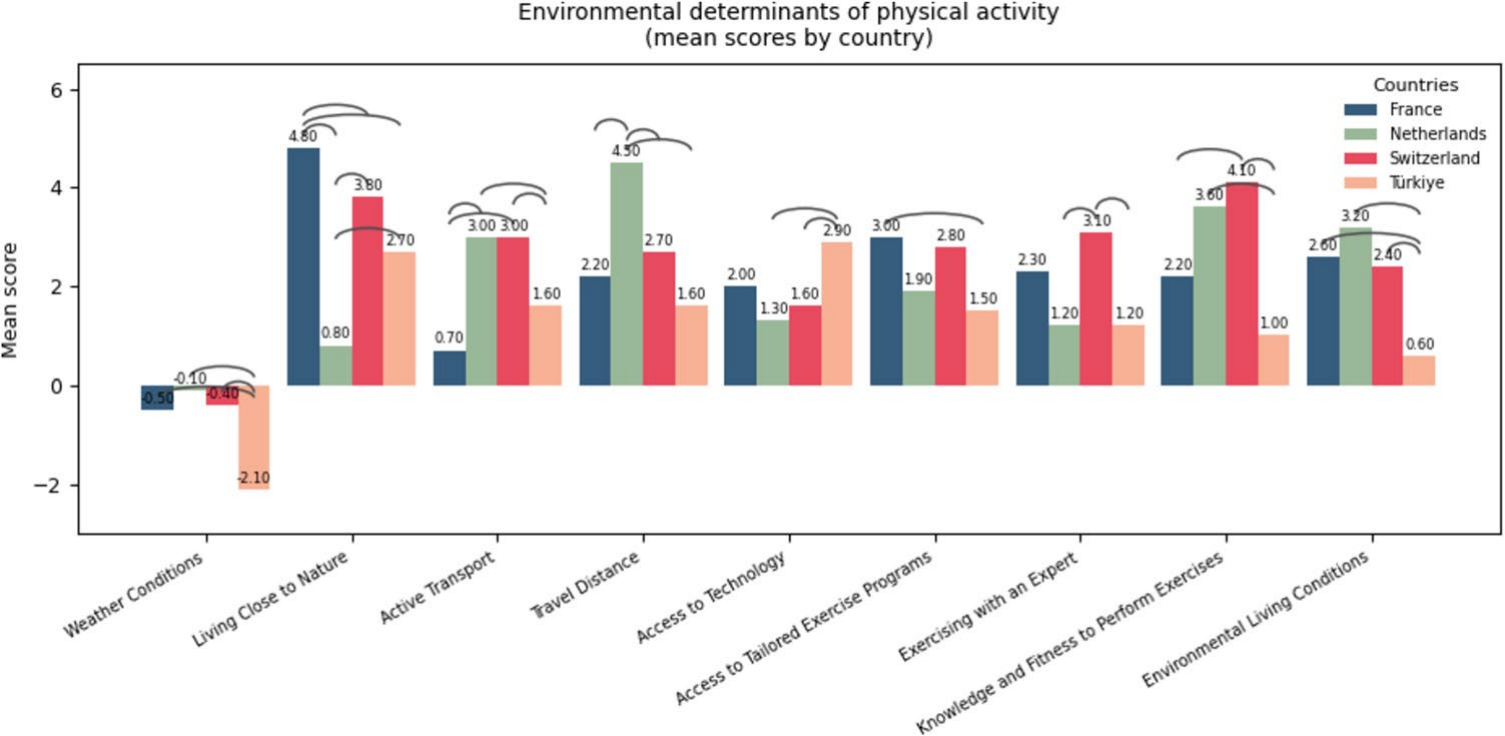
Mean scores of environmental determinants of PA across four countries (France, Switzerland, the Netherlands, Turkey), illustrating between-country differences for each item. Bars represent country-specific mean ratings for each environmental factor, with higher scores indicating stronger perceived facilitators and lower or negative scores indicating stronger perceived barriers. The arcs positioned above each item denote pairwise between-country differences

**Fig. 3:**
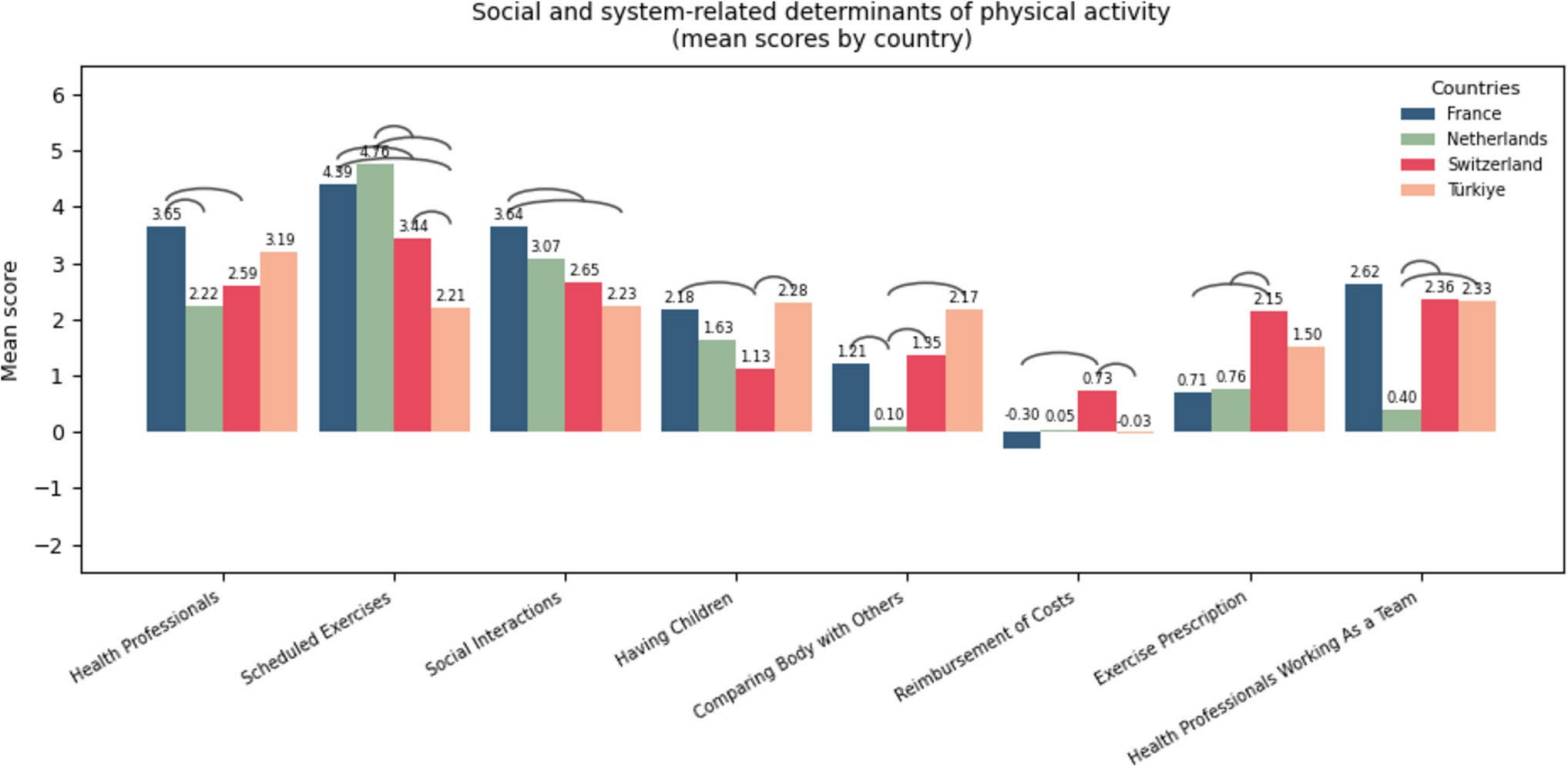
Mean scores of social and system-level determinants of PA across four countries (France, Switzerland, the Netherlands, Turkey), illustrating between-country differences for each item. Bars represent country-specific mean ratings for each environmental factor, with higher scores indicating stronger perceived facilitators and lower or negative scores indicating stronger perceived barriers. The arcs positioned above each item denote pairwise between-country differences

The statistically significant mean item scores (ranged from − 10 to 10) were presented in Figs. 2 and 3 displaying between-country differences. Non-significant items were presented in the Supplementary File 2. “Public transport that connected me with sports facilities” item from environmental domain was unsignificant after performing post-hoc analysis. Color-coded arcs were placed to illustrate the differences observed between countries. As an example, to read the Fig. 3, “health professionals” were reported as a stronger facilitator in France compared to the Netherlands and Switzerland.

**Fig. 4:**
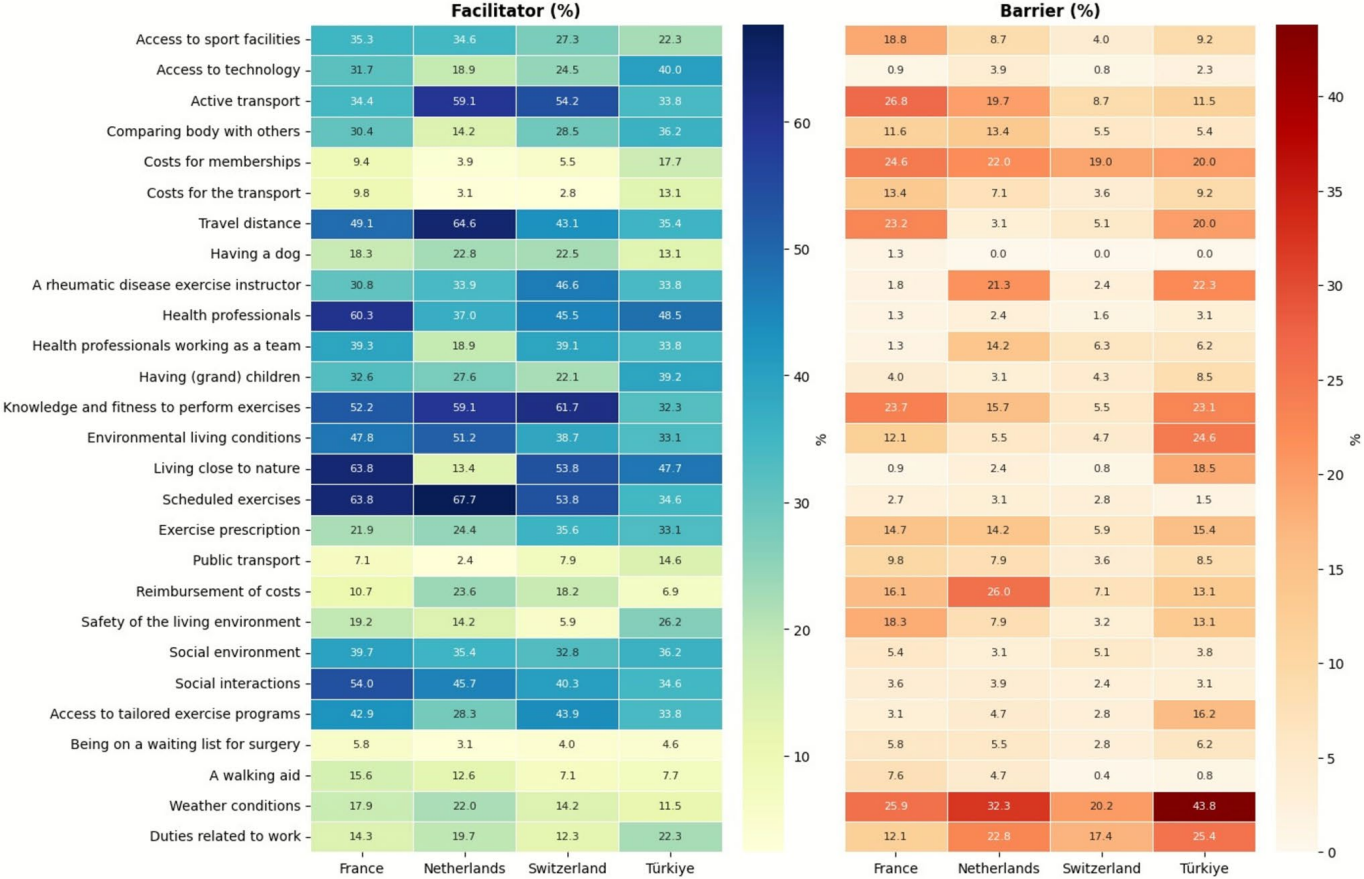
Facilitator (left) and barrier (right) percentages for each PA determinant across four countries. Higher color intensity indicates a greater proportion of participants endorsing that item as a facilitator or barrier

Figure 4 further illustrates the proportion of participants identifying each item as a facilitator or barrier to PA across countries. In Switzerland, the main facilitators were “knowledge and fitness to perform exercises,” “living close to nature,” and “active transport.” In the Netherlands, “scheduled exercises,” “travel distance to sport facilities,” “knowledge and fitness to perform exercises,” and “environmental living conditions” were most prominent. In France, “scheduled exercises,” “living close to nature,” and “health professionals” were predominant. In Turkey, “health professionals” and “living close to nature” were the main facilitators. With respect to barriers, “weather conditions” was mostly reported, particularly in Turkey, and to a lesser extent, the Netherlands. “Duties related to work” also emerged as a barrier, especially among participants in Turkey. Additionally, “cost for memberships to sport facilities or equipment” was frequently cited, most prominently in France.

## Discussion

This study provides novel insights into social, environmental, and system-level barriers and facilitators of PA among individuals with RMDs, revealing both common and different patters across four European countries. Common facilitators were scheduled exercise, knowledge and fitness to perform PA, and professional support. Common barriers were weather conditions, costs, and work demand.

However, social, environmental and system-level determinants of PA differ in relevance across countries despite comparable overall scores. Thus, between-country comparisons highlight distinct patterns of facilitators and barriers to PA. Scheduled exercise, such as planned activities or weekly sports training, were identified as prominent facilitators of PA, particularly emphasized in France and the Netherlands. Kanavaki et al. highlighted that regular participation in exercise results from the dynamic interplay of physical, personal, psychological, social, and environmental factors [12]. Engaging in regular PA may require individuals to set activity goals, monitor their progress, and exercise self-control through strategies such as problem solving [24]. Promoting structured routines may support adherence to PA recommendations.

Participants from France and Turkey in our cohort considered the role of healthcare professionals as an encouraging factor for PA, suggesting that individuals with arthritis value their advice [25]. Health professionals are key motivators, primarily by informing patients about the benefits of PA, yet this education often lacks of consistency and clarity [26]. Additional barriers to PA include insufficient information, limited referral to programs, and underuse of exercise prescription [25]. A study in France recommended integrating patient-healthcare professional communication and shared decision-making into education programs [27]. Conversely, contradictory statements from healthcare professionals, as well as lack of trust, have been linked to low confidence in professional expertise and insufficient teamwork [28]. These findings emphasize the importance of consistent communication and tailored guidance. French participants also regarded access to individualized exercise programs as a facilitator, yet the lack of RMD-specific programs remain an unmet need by patients [25]. However, individuals with RMDs lack of tailored exercise or PA advice from healthcare providers [29], highlighting the necessity for structured, disease-specific exercise guidance within routine care.

Participants from France, Switzerland, and Turkey commonly perceived living close to nature as a facilitator of PA. Besides this, participants from the Netherlands perceived proximity to a sport facility or nature as an encouraging factor. The World Health Organization’s Global Action Plan on Physical Activity 2018–2030 highlights green and blue spaces as key supportive environments for PA [30]. Proximity to parks and urban greenspaces is linked to higher PA participation, though their impact may depend on activity type [31, 32]. Our findings suggest that the built and natural environment can actively shape health behaviors, highlighting the need for urban planning and healthcare strategies that integrate nature and sport facilities into daily living contexts.

Weather conditions were perceived as a hindering factor for PA particularly by individuals from Turkey and the Netherlands. Weather—including hot and cold weather as well as rain—was the most common environmental barrier cited by patients with OA and RA in a previous study [25]. Weather conditions were also among the top three barriers to PA in patients with RA and axSpA [33]. In addition to cold or rainy weather [25], fluctuations in barometric pressure can aggravate symptoms, especially under extreme conditions [34]. The finding that weather emerged as a prominent barrier in our cohort is unsurprising considering the target population.

Active travel for transportation emerged as a key facilitator of PA in Switzerland and the Netherlands in our study. Active travel includes walking, cycling, and within-neighborhood walking, and is directly influenced by environmental features such as cycling infrastructure, walkability, safety, and degree of urbanization [35]. In Switzerland, walking is the predominant form of active travel, whereas in the Netherlands cycling is more common [36]. In another study, 41% to 55% of Dutch people met recommended PA levels by engaging in 24 to 28 min per day of walking or cycling [37]. This may be partly explained by supportive cultural norms toward active travel, as well as widespread use of public transport combined with walking or cycling in these countries [36]. Since active traveling provide many health benefits [38], policymakers should encourage active travel to promote PA in people with RMDs.

Knowing one’s capabilities and having sufficient fitness to perform activities or exercises emerged as an important facilitator factor for PA participation across countries, particularly in the Netherlands and Switzerland. A study from Scotland emphasized that patients’ knowledge and capacity to adapt their activity according to their physical condition was a determinant of PA [39]. Similarly, intention, motivation, confidence, and self-discipline were described as key facilitators by Swiss patients who participated in an osteoarthritis-specific exercise and education program [40].

Swiss participants identified “exercising with an expert in rheumatic diseases” and “access to tailored exercise programs” as important facilitators of PA. Individualized exercise and education programs under supervision have shown benefits, particularly in Switzerland [41]. Additionally, arthritis-specific programs help patients achieve recommended PA levels [41]. Therefore, the importance of professional supervision, peer-support in group settings, and access to individualized exercise programs should be emphasized in Switzerland. To optimize PA implementation across healthcare systems, such individualized programs should be encouraged and expanded in all countries.

In our sample, French participants specifically cited the cost of sport facilities or equipment as a hindering factor for PA participation. The cost of programs or equipment emerged as a barrier to exercise among the French population, consistent with previous reports that identified cost as a barrier among non-exerciser adults [25]. Likewise, OA patients reported the cost exercise materials as an obstacle to PA [42]. Financial constraints appear to particularly affect individuals with lower socioeconomic status, limiting their opportunities to engage in PA. Publicly funded or community-based programs may help mitigate these barriers.

A major strength of our study is the comprehensive quantification of barriers and facilitators to PA within environmental, social, and system-level contexts, moving beyond the predominant focus on individual determinants. Importantly, we compared these factors across four European countries in patients with RMDs, providing novel cross-country insights. By addressing these contextual determinants in an international sample, our study offers unique insights that can inform both clinical practice and policy development.

The study had several limitations that should be considered when interpreting the results. At first, we did not collect information on whether participants resided in urban or rural areas, which could influence environmental opportunities for PA. Moreover, the sample size per country was not intended for confirmatory psychometric validation or formal testing of measurement invariance, which will require larger and more balanced samples. Additionally, data were obtained from both clinical settings and online/social media recruitment channels, which may have introduced selection bias by overrepresenting individuals with higher health literacy, digital access, or stronger interest in PA. Furthermore, facilitators were more often reported than barriers, which may reflect that facilitators are generally perceived as more meaningful and personally relevant, or alternatively, some participants responded in a socially desirable way rather than with complete accuracy. Lastly, country-level differences may partly reflect demographic or disease-related composition of the participating samples, possibly due to recruiting different strategies and should therefore be interpreted with caution. Taken together, these factors should be considered when interpreting our findings, as they may influence interpretation and generalizability of the results.

## Conclusion

This study systematically identified common and diverging social, environmental, and system-level determinants of PA behavior in individuals with RMDs across four European countries. Facilitators and barriers were both perceived differently across countries, reflecting different cultural norms and environmental infrastructures. Tailoring interventions to these country-specific contextual differences may be essential to enhance adherence to PA recommendations and to support the effective implementation of PA promotion policies across these four European countries. Further research is needed to determine whether strategies targeting these determinants lead to improvements in PA participation.

## Supplementary Information

Below is the link to the electronic supplementary material.

ESM 1 COPA-BFQ questionnaire (.docx) including the questionnaire used in the manuscript. (DOCX 34.7 KB)

ESM 2 Unpublished data showing the statistical comparison of countries (.docx). (DOCX 42.9 KB)

ESM 3 CHERRIES checklist (.docx) including the checklist used in the study. (DOCX 29.6 KB)

## Data Availability

The datasets used and/or analyzed during the current study are available from the corresponding author on reasonable request.
