# Supplementary material for: Exploration of country-specific barriers and facilitators for the implementation of physical activity according to the EULAR physical activity recommendations for people with rheumatic musculoskeletal diseases in four different European countries: the COPA project
Source: Clin Rheumatol. 2026 Feb 16;45(4):2209–18. doi: 10.1007/s10067-026-07984-5 (PMC12979352; doi:10.1007/s10067-026-07984-5)
Supplement: Supplementary file 1 — COPA-BFQ questionnaire (.docx) including the questionnaire used in the manuscript. (DOCX 34.7 KB) [file 10067_2026_7984_MOESM1_ESM.docx]

**Exploration of Country-specific Barriers and Facilitators for the Implementation of Physical Activity According to the EULAR Physical Activity Recommendations for People with Rheumatic Musculoskeletal Diseases in Four Different European Countries: the COPA project**

**Journal Name: Clinical Rheumatology**

Özgül Öztürk, Assoc. Prof. ^1^, David Ueckert, BA/BSc ^2^, Leti van Bodegom-Vos, Assoc. Prof ^3^, Salima van Weely, PhD ^4, 5^, Özlem Feyzioğlu, Assoc. Prof. ^1^, Karin Niedermann, Professor ^6^, Anne-Kathrin Rausch Osthoff, PhD ^6^, Thomas Davergne, Assist. Prof. ^7^

^1^ Acıbadem University, Physiotherapy and Rehabilitation, Istanbul, Türkiye

^2^ Leiden University Medical Center, Department of Physiotherapy, Leiden, Netherlands

^3^ Leiden University Medical Center, Department of Biomedical Data Sciences, Leiden, Netherlands

^4^ HU University of Applied Sciences, Institute of Allied Health Professions, Utrecht, Netherlands

^5^ Leiden University Medical Center, Department of Orthopedics, Rehabilitation and Physical Therapy, Leiden, Netherlands

^6^ Zurich University of Applied Sciences, School of Health Sciences, Institute of Physiotherapy, Zurich, Switzerland

^7^ Université Paris Cité and Université Sorbonne Paris Nord, Inserm, INRAE, Center for Research in Epidemiology and Statistics (CRESS), F-75004 Paris, France

**Corresponding Author:**

Özgül Öztürk, PT, Associate Professor

**Address:** Acıbadem University, Kayışdağı St, No:32, 34752, Ataşehir/İstanbul, Türkiye

**Phone:** +90 216 500 41 85

**Email:** ozgul.ozturk@acibadem.edu.tr

***COPA-BFQ Questionnaire***

***Dear participant,***

***Thank you for supporting our research in physical activity promotion. We would like to know which factors are barriers (hindering/limiting) or facilitators (supportive/promoting/enabling) for you being physically active.***

***According to the World Health Organisation physical activity is defined as any bodily movement produced by skeletal muscles that requires energy expenditure. Physical activity refers to all movement including during leisure time (e.g. exercises/sports), for transport to get to and from places, or as part of a person’s work. Both moderate- and vigorous-intensity physical activity improve health.***

***Before we dive into barriers and facilitators for being physically active, we would like to know more about your physical activity behavior. All answers are anonymized – that means the answers cannot be traced back to you.***

1. ***On average, how many days per week do you engage in moderate to vigorous physical activity (like brisk walking)? 0-7 days***
2. ***On average, how many minutes in total do you engage in physical activity at this level? _____ minutes***
3. ***How many days per week do you perform muscle strengthening exercises, such as bodyweight exercises or resistance training? 0-7 days***

***Thank you.***

***In answering the next set of questions, please think about your physical activity behavior during the last 4 weeks. Please indicate for each item if it had enabled you, prevented you, or did not have an impact on your being physically active during the previous 4 weeks. Next, if you decided the item had an impact on your physical activity, please rate the importance of that. All answers are anonymized, the answers cannot be traced back to you.***

|  | *Online survey*  *Step 1: decide if the item was a barrier or a facilitator or none during the last 4 weeks.*  *Step 2: quantify the impact (except if you quote “have no impact on my physical activity”).* | | | | | | | | | | | | | |
| --- | --- | --- | --- | --- | --- | --- | --- | --- | --- | --- | --- | --- | --- | --- |
| Social factors | | | | | | | | | | | | | | |
| My social environment (family, friends, colleagues at work)  □ encouraged me to be physically active.  □ prevented me from being physically active.  □ had no impact on my physical activity. | | Not at all | 0 | 1 | 2 | 3 | 4 | 5 | 6 | 7 | 8 | 9 | 10 | Very much (fac)  A lot (barrier) |
| Health Professionals (e.g. doctor, nurse, physiotherapist)  □ encouraged me to be physically active.  □ prevented me from being physical active.  □ had no impact on my physical activity. | | Not at all | 0 | 1 | 2 | 3 | 4 | 5 | 6 | 7 | 8 | 9 | 10 | Very much (fac)  A lot (barrier) |
| Scheduled exercises (e.g. planned activities, weekly sports training)  □ encouraged me to be physically active.  □ prevented me from being physically active.  □ had no impact on my physical activity. | | Not at all | 0 | 1 | 2 | 3 | 4 | 5 | 6 | 7 | 8 | 9 | 10 | Very much (fac)  A lot (barrier) |
| Having social interactions during activity (e.g. with exercise partner, group setting)  □ encouraged me to be physically active.  □ prevented me from being physically active, I preferred to exercise alone.  □ has no impact on my physical activity. | | Not at all | 0 | 1 | 2 | 3 | 4 | 5 | 6 | 7 | 8 | 9 | 10 | Very much (fac)  A lot (barrier) |
| Having a dog (or another animal to walk with)  □ encouraged me to be physically active.  □ prevented me from being physical active.  □ I do not have a dog. | | Not at all | 0 | 1 | 2 | 3 | 4 | 5 | 6 | 7 | 8 | 9 | 10 | Very much (fac)  A lot (barrier) |
| Having (grand) children  □ encouraged me to be physically active.  □ prevented me from being physical active.  □ I do not have (grand) children. | | Not at all | 0 | 1 | 2 | 3 | 4 | 5 | 6 | 7 | 8 | 9 | 10 | Very much (fac)  A lot (barrier) |
| Comparing my body with others regarding appearance or fitness level  □ encouraged me to be physically active.  □ prevented me from being physically active.  □ had no impact on my physical activity. | | Not at all | 0 | 1 | 2 | 3 | 4 | 5 | 6 | 7 | 8 | 9 | 10 | Very much (fac)  A lot (barrier) |
| Environmental factors | | | | | | | | | | | | | | |
| Weather conditions in the region I live in  □ encouraged me to be physically active.  □ prevented me from being physical active.  □ had no impact on my physical activity. | | Not at all | 0 | 1 | 2 | 3 | 4 | 5 | 6 | 7 | 8 | 9 | 10 | Very much (fac)  A lot (barrier) |
| Safety of my living environment (e.g. streetlight, bike lane)  □ encouraged me to be physically active.  □ prevented me from being physical active.  □ had no impact on my physical activity. | | Not at all | 0 | 1 | 2 | 3 | 4 | 5 | 6 | 7 | 8 | 9 | 10 | Very much (fac)  A lot (barrier) |
| Living close to nature  □ encouraged me to be physically active.  □ prevented me from being physically active.  □ had no impact on my physical activity. | | Not at all | 0 | 1 | 2 | 3 | 4 | 5 | 6 | 7 | 8 | 9 | 10 | Very much (fac)  A lot (barrier) |
| Public Transport that connected me with sports facilities,  □ encouraged me to be physically active.  □ prevented me from being physical active.  □ had no impact on my physical activity. | | Not at all | 0 | 1 | 2 | 3 | 4 | 5 | 6 | 7 | 8 | 9 | 10 | Very much (fac)  A lot (barrier) |
| Active Transport, e.g. biking, stair climbing.  □ encouraged me to be physically active.  □ is difficult for me and, therefore, limited me being physically active.  □ had no impact on my physical activity. | | Not at all | 0 | 1 | 2 | 3 | 4 | 5 | 6 | 7 | 8 | 9 | 10 | Very much (fac)  A lot (barrier) |
| Access to sports facilities (e.g. public swimming pool)  □ encouraged me to be physically active.  □ the lack of access prevented me from being physical active.  □ had no impact on my physical activity. | | Not at all | 0 | 1 | 2 | 3 | 4 | 5 | 6 | 7 | 8 | 9 | 10 | Very much (fac)  A lot (barrier) |
| Travel distance  □ I live close to a sport facility/nature (walking <1km, biking <3km, car <15km) /travel time (10-20Min), this helped me to be physically active.  □ I live far from a sport facility/nature (walking >1km, biking >3km, car >15km) /travel time (20> Min), this limited me to be physically active.  □ had no impact on my physical activity. | | Not at all | 0 | 1 | 2 | 3 | 4 | 5 | 6 | 7 | 8 | 9 | 10 | Very much (fac)  A lot (barrier) |
| Access to websites or smartphone applications for instructions or monitoring physical activity  □ encouraged me being physically active.  □ prevented me from being physically active.  □ had no impact on my physical activity. | | Not at all | 0 | 1 | 2 | 3 | 4 | 5 | 6 | 7 | 8 | 9 | 10 | Very much (fac)  A lot (barrier) |
| Access to exercise programs designed for me and adapted for my condition  □ encouraged me to be physically active.  □ prevented me being physically active.  □ had no impact on my physical activity. | | Not at all | 0 | 1 | 2 | 3 | 4 | 5 | 6 | 7 | 8 | 9 | 10 | Very much (fac)  A lot (barrier) |
| An exercise instructor who is expert in rheumatic musculoskeletal diseases  □ supervising my exercises, encouraged me being physically active.  □ was not available, which was hindering me being physically active.  □ had no impact on my physical activity. | | Not at all | 0 | 1 | 2 | 3 | 4 | 5 | 6 | 7 | 8 | 9 | 10 | Very much (fac)  A lot (barrier) |
| Knowledge and fitness to perform by my own/not supervised exercises  □ I know how to exercise on my own, this encouraged me being physically active.  □ I do not know which exercises are good for me; this was hindering me being physically active.  □ had no impact on my physical activity. | | Not at all | 0 | 1 | 2 | 3 | 4 | 5 | 6 | 7 | 8 | 9 | 10 | Very much (fac)  A lot (barrier) |
| A walking aid  □ having a walking aid helped me being physically active.  □ the lack of a walking aid prevented me being physically active.  □ had no impact on my physical activity. | | Not at all | 0 | 1 | 2 | 3 | 4 | 5 | 6 | 7 | 8 | 9 | 10 | Very much (fac)  A lot (barrier) |
| My environmental living conditions, (e.g. number of steps in my house, having a garden, steep mountains in the region)  □ encouraged me to be physically active.  □ prevented me being physically active.  □ had no impact on my physical activity. | | Not at all | 0 | 1 | 2 | 3 | 4 | 5 | 6 | 7 | 8 | 9 | 10 | Very much (fac)  A lot (barrier) |
| System factors | | | | | | | | | | | | | | |
| Reimbursement of costs from health assurance  □ receiving reimbursements encouraged me to be physically active.  □ the lack of reimbursements prevented me being physically active.  □ had no impact on my physical activity. | | Not at all | 0 | 1 | 2 | 3 | 4 | 5 | 6 | 7 | 8 | 9 | 10 | Very much (fac)  A lot (barrier) |
| Costs for the transport to sports facilities  □ low costs encouraged me to be physically active.  □ high costs prevented me being physically active.  □ had no impact on my physical activity. | | Not at all | 0 | 1 | 2 | 3 | 4 | 5 | 6 | 7 | 8 | 9 | 10 | Very much (fac)  A lot (barrier) |
| Costs for memberships or entrance to sport facilities or sport devices  □ low costs encouraged me to be physically active.  □ high costs prevented me being physically active.  □ had no impact on my physical activity. | | Not at all | 0 | 1 | 2 | 3 | 4 | 5 | 6 | 7 | 8 | 9 | 10 | Very much (fac)  A lot (barrier) |
| Duties related to work (paid work or unpaid care work, such as care or volunteering)  □ encouraged me to be physically active.  □ prevented me being physically active.  □ had no impact on my physical activity. | | Not at all | 0 | 1 | 2 | 3 | 4 | 5 | 6 | 7 | 8 | 9 | 10 | Very much (fac)  A lot (barrier) |
| Exercise prescription  □ having an exercise prescription encouraged me to be physically active.  □ the lack of an exercise prescription prevented me being physically active.  □ had no impact on my physical activity. | | Not at all | 0 | 1 | 2 | 3 | 4 | 5 | 6 | 7 | 8 | 9 | 10 | Very much (fac)  A lot (barrier) |
| Health professionals working as a team (e.g. doctors, nurses, physiotherapists)  □ encouraged me to be physically active.  □ the lack of interdisciplinary work of health professionals prevented me being physically active.  □ had no impact on my physical activity. | | Not at all | 0 | 1 | 2 | 3 | 4 | 5 | 6 | 7 | 8 | 9 | 10 | Very much (fac)  A lot (barrier) |
| Being on a waiting list for surgery  □ encouraged me to be physically active.  □ prevented me being physically active.  □ had no impact on my physical activity. | | Not at all | 0 | 1 | 2 | 3 | 4 | 5 | 6 | 7 | 8 | 9 | 10 | Very much (fac)  A lot (barrier) |
