# Supplementary material for: Exploration of country-specific barriers and facilitators for the implementation of physical activity according to the EULAR physical activity recommendations for people with rheumatic musculoskeletal diseases in four different European countries: the COPA project
Source: Clin Rheumatol. 2026 Feb 16;45(4):2209–18. doi: 10.1007/s10067-026-07984-5 (PMC12979352; doi:10.1007/s10067-026-07984-5)
Supplement: Supplementary file 2 — Unpublished data showing the statistical comparison of countries (.docx). (DOCX 42.9 KB) [file 10067_2026_7984_MOESM2_ESM.docx]

**Exploration of Country-specific Barriers and Facilitators for the Implementation of Physical Activity According to the EULAR Physical Activity Recommendations for People with Rheumatic Musculoskeletal Diseases in Four Different European Countries: the COPA project**

**Journal Name: Clinical Rheumatology**

Özgül Öztürk, Assoc. Prof. ^1^, David Ueckert, BA/BSc ^2^, Leti van Bodegom-Vos, Assoc. Prof ^3^, Salima van Weely, PhD ^4, 5^, Özlem Feyzioğlu, Assoc. Prof. ^1^, Karin Niedermann, Professor ^6^, Anne-Kathrin Rausch Osthoff, PhD ^6^, Thomas Davergne, Assist. Prof. ^7^

^1^ Acıbadem University, Physiotherapy and Rehabilitation, Istanbul, Türkiye

^2^ Leiden University Medical Center, Department of Physiotherapy, Leiden, Netherlands

^3^ Leiden University Medical Center, Department of Biomedical Data Sciences, Leiden, Netherlands

^4^ HU University of Applied Sciences, Institute of Allied Health Professions, Utrecht, Netherlands

^5^ Leiden University Medical Center, Department of Orthopedics, Rehabilitation and Physical Therapy, Leiden, Netherlands

^6^ Zurich University of Applied Sciences, School of Health Sciences, Institute of Physiotherapy, Zurich, Switzerland

^7^ Université Paris Cité and Université Sorbonne Paris Nord, Inserm, INRAE, Center for Research in Epidemiology and Statistics (CRESS), F-75004 Paris, France

**Corresponding Author:**

Özgül Öztürk, PT, Associate Professor

**Address:** Acıbadem University, Kayışdağı St, No:32, 34752, Ataşehir/İstanbul, Türkiye

**Phone:** +90 216 500 41 85

**Email:** ozgul.ozturk@acibadem.edu.tr

Supplementary File 2

Table 1. Comparison of the mean score of each items between countries (from -10 to 10) (non-significant items).

| **Domain and questions, mean (SD)** | **All patients**  **(n = 734)** | **France**  **(n = 224)** | **Netherlands**  **(n = 127)** | **Switzerland**  **(n = 253)** | **P value** |
| --- | --- | --- | --- | --- | --- |
| ***Social*** |  |  |  |  |  |
| My social environment (family, friends, colleagues at work) | 2.00 (3.88) | 2.24 (3.84) | 1.68 (3.5) | 1.88 (3.94) | 0.487 |
| Having a dog (or another animal to walk with) | 1.25 (3.07) | 1.84 (3.47) | 1.89 (3.61) | 1.01 (2.74) | 0.060 |
| ***Environmental*** |  |  |  |  |  |
| Safety of my living environment (e.g. streetlight, bike lane) | 0.01 (4.22) | 0.63 (3.17) | 0.15 (1.91) | 1.11 (4.46) | 0.071 |
| Public transport that connected me with sports facilities | -0.27 (3.09) | -0.40 (2.36) | 0.22 (2.29) | 0.45 (3.34) | 0.022* |
| Access to sports facilities (e.g. public swimming pool) | 1.26 (5.49) | 2.03 (4.57) | 1.63 (3.69) | 1.2 (3.94) | 0.339 |
| A walking aid | 0.48 (3.44) | 0.71 (3.10) | 0.38 (1.61) | 0.44 (1.73) | 0.914 |
| ***System*** |  |  |  |  |  |
| Cost for transportation | -0.27 (3.43) | -0.34 (2.30) | -0.55 (1.73) | 0.28 (3.29) | 0.276 |
| Cost for membership | -1.19 (4.34) | -1.29 (3.44) | -0.99 (3.47) | -0.10 (4.61) | 0.098 |
| Duties related to work | 0.16 (4.00) | -0.23 (4.82) | -0.31 (4.00) | 0.01 (5.31) | 0.615 |
| Being at waiting list for surgery | 0.08 (2.45) | -0.04 (2.10) | 0.06 (2.12) | -0.20 (2.56) | 0.705 |

SD; standard deviation.

*Non-significant after post-hoc analysis (Tamhane).

Supplementary File 2

Table 2. Comparison of the percentage of people with RMDs perceving social factors as facilitators, barriers, or neutral to PA.

| **Items** | **Countries** | **Facilitator, n (%)** | **Barrier, n (%)** | **Neutral, n (%)** |
| --- | --- | --- | --- | --- |
| Social environment | France | 89 (39.7) | 12 (5.4) | 123 (54.9) |
|  | Netherlands | 45 (35.4) | 4 (3.1) | 78 (61.4) |
|  | Switzerland | 83 (32.8) | 13 (5.1) | 157 (62.1) |
|  | Türkiye | 47 (36.2) | 5 (3.8) | 78 (60.0) |
|  |  | 0.687 | | |
| Health professionals | France | 135 (60.3) | 3 (1.3) | 86 (38.4) |
|  | Netherlands | 47 (37.0) | 3 (2.4) | 77 (60.6) |
|  | Switzerland | 115 (45.5) | 4 (1.6) | 134 (53.0) |
|  | Türkiye | 63 (48.5) | 4 (3.1) | 63 (48.5) |
|  |  | **0.001** | | |
| Scheduled exercises | France | 143 (63.8) | 6 (2.7) | 75 (33.5) |
|  | Netherlands | 86 (67.7) | 4 (3.1) | 37 (29.1) |
|  | Switzerland | 136 (53.8) | 7 (2.8) | 110 (43.5) |
|  | Türkiye | 45 (34.6) | 2 (1.5) | 83 (63.8) |
|  |  | **0.001** | | |
| Social interactions | France | 121 (54.0) | 8 (3.6) | 95 (42.4) |
|  | Netherlands | 58 (45.7) | 5 (3.9) | 64 (50.4) |
|  | Switzerland | 102 (40.3) | 6 (2.4) | 145 (57.3) |
|  | Türkiye | 45 (34.6) | 4 (3.1) | 81 (62.3) |
|  |  | **0.008** | | |
| Having a dog | France | 41 (18.3) | 3 (1.3) | 180 (80.4) |
|  | Netherlands | 29 (22.8) | 0 (0) | 98 (77.2) |
|  | Switzerland | 57 (22.5) | 0 (0) | 196 (77.5) |
|  | Türkiye | 17 (13.1) | 0 (0) | 113 (86.9) |
|  |  | 0.063 | | |
| Having (grand) children | France | 73 (32.6) | 9 (4.0) | 142 (63.4) |
|  | Netherlands | 35 (27.6) | 4 (3.1) | 88 (69.3) |
|  | Switzerland | 56 (22.1) | 11 (4.3) | 186 (73.5) |
|  | Türkiye | 51 (39.2) | 11 (8.5) | 68 (52.3) |
|  |  | **0.002** | | |
| Comparing body with others | France | 68 (30.4) | 26 (11.6) | 130 (58.0) |
|  | Netherlands | 18 (14.2) | 17 (13.4) | 92 (72.4) |
|  | Switzerland | 72 (28.5) | 14 (5.5) | 167 (66.0) |
|  | Türkiye | 47 (36.2) | 7 (5.4) | 76 (58.5) |
|  |  | **<0.001** | | |

Bold values indicate significancy. Statistical comparison was performed by using chi-square or Fisher’s exact tests.

Supplementary File 2

Table 3. Comparison of the percentage of people with RMDs perceving environmental factors as facilitators, barriers, or neutral to PA.

| **Items** | **Countries** | **Facilitator, n (%)** | **Barrier, n (%)** | **Neutral, n (%)** |
| --- | --- | --- | --- | --- |
| Weather conditions | France | 40 (17.9) | 58 (25.9) | 126 (56.3) |
|  | Netherlands | 28 (22.0) | 41 (32.3) | 58 (45.7) |
|  | Switzerland | 36 (14.2) | 51 (20.2) | 166 (65.6) |
|  | Türkiye | 15 (11.5) | 57 (43.8) | 58 (44.6) |
|  |  | **<0.001** | | |
| Safety of the living environment | France | 43 (19.2) | 41 (18.3) | 140 (62.5) |
|  | Netherlands | 18 (14.2) | 10 (7.9) | 99 (78.0) |
|  | Switzerland | 15 (5.9) | 8 (3.2) | 230 (90.9) |
|  | Türkiye | 34 (26.2) | 17 (13.1) | 79 (60.8) |
|  |  | **<0.001** | | |
| Living close to nature | France | 143 (63.8) | 2 (0.9) | 79 (35.3) |
|  | Netherlands | 17 (13.4) | 3 (2.4) | 107 (84.3) |
|  | Switzerland | 136 (53.8) | 2 (0.8) | 115 (45.5) |
|  | Türkiye | 62 (47.7) | 24 (18.5) | 44 (33.8) |
|  |  | **<0.001** | | |
| Public transport | France | 16 (7.1) | 22 (9.8) | 186 (83.0) |
|  | Netherlands | 3 (2.4) | 10 (7.9) | 114 (89.8) |
|  | Switzerland | 19 (14.6) | 11 (8.5) | 100 (76.9) |
|  | Türkiye | 20 (7.9) | 9 (3.6) | 224 (88.5) |
|  |  | **<0.001** | | |
| Active transport | France | 77 (34.4) | 60 (26.8) | 87 (38.8) |
|  | Netherlands | 75 (59.1) | 25 (19.7) | 27 (21.3) |
|  | Switzerland | 137 (54.2) | 22 (8.7) | 94 (37.2) |
|  | Türkiye | 44 (33.8) | 15 (11.5) | 71 (54.6) |
|  |  | <0.001* | | |
| Access to sports facilities | France | 79 (35.3) | 42 (18.8) | 103 (46.0) |
|  | Netherlands | 44 (34.6) | 11 (8.7) | 72 (56.7) |
|  | Switzerland | 69 (27.3) | 10 (4.0) | 174 (68.8) |
|  | Türkiye | 29 (22.3) | 12 (9.2) | 89 (68.5) |
|  |  | **<0.001** | | |
| Travel distance | France | 110 (49.1) | 52 (23.2) | 62 (27.7) |
|  | Netherlands | 82 (64.6) | 4 (3.1) | 41 (32.3) |
|  | Switzerland | 109 (43.1) | 13 (5.1) | 131 (51.8) |
|  | Türkiye | 46 (35.4) | 26 (20.0) | 58 (44.6) |
|  |  | **<0.001** | | |
| Access to technology | France | 71 (31.7) | 2 (0.9) | 151 (67.4) |
|  | Netherlands | 24 (18.9) | 5 (3.9) | 98 (77.2) |
|  | Switzerland | 62 (24.5) | 2 (0.8) | 189 (74.7) |
|  | Türkiye | 52 (40.0) | 3 (2.3) | 75 (57.7) |
|  |  | **<0.001** | | |
| Access to tailored exercise programs | France | 96 (42.9) | 7 (3.1) | 121 (54.0) |
|  | Netherlands | 36 (28.3) | 6 (4.7) | 85 (66.9) |
|  | Switzerland | 111 (43.9) | 7 (2.8) | 135 (53.4) |
|  | Türkiye | 44 (33.8) | 21 (16.2) | 65 (50.0) |
|  |  | **<0.001** | | |
| A rheumatic disease exercise instructor | France | 69 (30.8) | 4 (1.8) | 151 (67.4) |
|  | Netherlands | 43 (33.9) | 27 (21.3) | 57 (44.9) |
|  | Switzerland | 118 (46.6) | 6 (2.4) | 129 (51.0) |
|  | Türkiye | 44 (33.8) | 29 (22.3) | 57 (43.8) |
|  |  | **<0.001** | | |
| Knowledge and fitness to perform exercises | France | 117 (52.2) | 53 (23.7) | 54 (24.1) |
|  | Netherlands | 75 (59.1) | 20 (15.7) | 32 (25.2) |
|  | Switzerland | 156 (61.7) | 14 (5.5) | 83 (32.8) |
|  | Türkiye | 42 (32.3) | 30 (23.1) | 58 (44.6) |
|  |  | <0.001* | | |
| Walking aid | France | 35 (15.6) | 17 (7.6) | 172 (76.8) |
|  | Netherlands | 16 (12.6) | 6 (4.7) | 105 (82.7) |
|  | Switzerland | 18 (7.1) | 1 (0.4) | 234 (92.5) |
|  | Türkiye | 10 (7.7) | 1 (0.8) | 119 (91.5) |
|  |  | <0.001* | | |
| Environmental living conditions | France | 107 (47.8) | 27 (12.1) | 90 (40.2) |
|  | Netherlands | 65 (51.2) | 7 (5.5) | 55 (43.3) |
|  | Switzerland | 98 (38.7) | 12 (4.7) | 143 (56.5) |
|  | Türkiye | 43 (33.1) | 32 (24.6) | 55 (42.3) |
|  |  | <0.001* | | |

Bold values indicate significancy. Statistical comparison was performed by using chi-square or Fisher’s exact tests.

Supplementary File 2

Table 4. Comparison of the percentage of people with RMDs perceving system-level factors as facilitators, barriers, or neutral to PA.

| **Items** | **Countries** | **Facilitator, n (%)** | **Barrier, n (%)** | **Neutral, n (%)** |
| --- | --- | --- | --- | --- |
| Reimbursement of costs | France | 24 (10.7) | 36 (16.1) | 164 (73.2) |
|  | Netherlands | 30 (23.6) | 33 (26.0) | 64 (50.4) |
|  | Switzerland | 46 (18.2) | 18 (7.1) | 189 (74.7) |
|  | Türkiye | 9 (6.9) | 17 (13.1) | 104 (80.0) |
|  |  | | **<0.001** | |
| Costs for transport | France | 22 (9.8) | 30 (13.4) | 172 (76.8) |
|  | Netherlands | 4 (3.1) | 9 (7.1) | 114 (89.8) |
|  | Switzerland | 7 (2.8) | 9 (3.6) | 237 (93.7) |
|  | Türkiye | 17 (13.1) | 12 (9.2) | 101 (77.7) |
|  |  | | **<0.001** | |
| Cost for membership | France | 21 (9.4) | 55 (24.6) | 148 (66.1) |
|  | Netherlands | 5 (3.9) | 28 (22.0) | 94 (74.0) |
|  | Switzerland | 14 (5.5) | 48 (19.0) | 191 (75.5) |
|  | Türkiye | 23 (17.7) | 26 (20.0) | 81 (62.3) |
|  |  | | **<0.001** | |
| Duties related to work | France | 32 (14.3) | 27 (12.1) | 165 (73.7) |
|  | Netherlands | 25 (19.7) | 29 (22.8) | 73 (57.5) |
|  | Switzerland | 31 (12.3) | 44 (17.4) | 178 (70.4) |
|  | Türkiye | 29 (22.3) | 33 (25.4) | 68 (52.3) |
|  |  | | **<0.001** | |
| Exercise prescription | France | 49 (21.9) | 33 (14.7) | 142 (63.4) |
|  | Netherlands | 31 (24.4) | 18 (14.2) | 78 (61.4) |
|  | Switzerland | 90 (35.6) | 15 (5.9) | 148 (58.5) |
|  | Türkiye | 43 (33.1) | 20 (15.4) | 67 (51.5) |
|  |  | | **0.001** | |
| Health professionals working as a team | France | 88 (39.3) | 3 (1.3) | 133 (59.4) |
|  | Netherlands | 24 (18.9) | 18 (14.2) | 85 (66.9) |
|  | Switzerland | 99 (39.1) | 16 (6.3) | 138 (54.5) |
|  | Türkiye | 44 (33.8) | 8 (6.2) | 78 (60.0) |
|  |  | | **0.001** | |
| Being on a waiting list for surgery | France | 13 (5.8) | 13 (5.8) | 198 (88.4) |
|  | Netherlands | 4 (3.1) | 7 (5.5) | 116 (91.3) |
|  | Switzerland | 10 (4.0) | 7 (2.8) | 236 (93.3) |
|  | Türkiye | 6 (4.6) | 8 (6.2) | 116 (89.2) |
|  |  | | 0.520 | |

Bold values indicate significancy. Statistical comparison was performed by using chi-square or Fisher’s exact tests.
